# PEEK in modern maxillofacial surgery: clinical applications, limitations and future opportunities

**DOI:** 10.1038/s41415-026-9789-1

**Published:** 2026-07-10

**Authors:** Betul Gedik, Mustafa Ayhan, Abdulkadir Burak Cankaya, Mehmet Ali Erdem

**Affiliations:** Department of Oral and Maxillofacial Surgery, Faculty of Dentistry, Istanbul University, Istanbul, 34116, Turkey

## Abstract

**Aims** To provide a contemporary, clinically oriented overview of polyetheretherketone (PEEK) as a biomaterial in oral and maxillofacial surgery, focusing on its mechanical properties, clinical applications and future potential in everyday dental practice.

**Methods** A narrative literature review was conducted using PubMed, Scopus and Web of Science to identify experimental, biomechanical and clinical studies on PEEK and carbon fibre-reinforced PEEK in craniomaxillofacial and dental applications. Particular emphasis was placed on material properties, patient-specific implant technology, osteosynthesis, temporomandibular joint (TMJ) prostheses and dental implant–related uses, as well as recent developments in surface modification and additive manufacturing.

**Results** PEEK exhibits an elastic modulus closer to cortical bone than titanium, radiolucency and high fatigue resistance, making it attractive for patient-specific reconstruction plates, orbital and mandibular implants, TMJ components and prosthetic frameworks. However, its inherently bioinert surface limits osteointegration, necessitating surface engineering strategies such as plasma treatment, nanotopography and bioactive coatings to enhance bone–implant contact.

**Conclusions** PEEK and its composites are poised to play an expanding role in metal-free, digitally planned reconstruction in oral and maxillofacial surgery. Translation into routine dental implantology will depend on robust clinical data confirming long-term osseointegration and mechanical reliability.

## Key points

- Polyetheretherketone (PEEK) possesses bone-like elastic properties and radiolucency, making it an attractive material for anatomical reconstruction while facilitating post-operative imaging evaluation.
- Patient-specific PEEK implants provide improved adaptation to defect morphology, resulting in enhanced fit, contour accuracy, and more reliable post-operative assessment.
- Further advances in surface modification technologies are crucial to enhance the osteointegration potential of PEEK and support its long-term clinical performance.

## Introduction

The field of maxillofacial surgery increasingly relies on biomaterials capable of restoring complex anatomical structures under high functional demands. Historically, titanium has served as the principal material due to its high strength, corrosion resistance, and proven biocompatibility. However, titanium's limitations have become more prominent as surgical expectations evolve toward personalised reconstruction, long-term imaging clarity, improved aesthetic outcomes, and reduced biological reactivity.^1^ Titanium's elastic modulus far exceeds that of cortical bone, resulting in stress shielding, altered load transmission, and possible bone resorption. Additionally, its radiopacity complicates postoperative imaging, creating artefacts that obscure tumour surveillance, fracture healing, or implant positioning.

These limitations prompted interest in alternative materials that could better mimic bone biomechanics and enable seamless integration with modern digital workflows. PEEK (polyetheretherketone), a high-performance polymer first used in aerospace industries, entered medicine through orthopaedic and spinal applications, where its radiolucency and bone-like elasticity demonstrated significant clinical potential.^2^ Over the past decade, advances in computer-aided design/computer-aided manufacturing (CAD/CAM) technology, virtual surgical planning, and 3D printing have profoundly accelerated PEEK's adaptation to maxillofacial surgery, allowing fabrication of highly precise patient-specific implants with superior anatomical fidelity.^3^

In clinical practice, the applications of PEEK can be broadly categorised into two distinct domains: surgical use in oral and maxillofacial reconstruction and temporomandibular joint (TMJ) prosthetics, and prosthetic or dental applications, such as implant abutments, temporary frameworks and experimental implant bodies. These domains differ markedly in their biomechanical requirements, biological interactions, and clinical outcome measures. Accordingly, the primary focus of this review is on PEEK as a surgical and reconstructive material in maxillofacial and TMJ applications, while dental and prosthetic uses are addressed in a limited and contextual manner. This structured approach aims to ensure conceptual clarity and alignment between the scope of the review and the available clinical evidence.

## Material science and biological foundations

### Chemical composition and thermo-mechanical structure

PEEK belongs to the family of polyaryletherketone polymers and is characterised by its semi-crystalline structure composed of rigid aromatic rings connected through ether and ketone linkages.^4^ This chemical configuration provides a balance of toughness, thermal stability, and rigidity. The crystalline regions of PEEK contribute to high mechanical strength and resistance to deformation, while amorphous zones enhance flexibility and fatigue tolerance.^5^ Together, these features allow PEEK to maintain structural integrity under fluctuating masticatory loads, making it highly suitable for craniomaxillofacial biomechanics.

From a thermal perspective, PEEK demonstrates an exceptionally high melting point of approximately 343 °C, allowing the material to withstand autoclave sterilisation and thermal fluctuations without performance degradation.^6^ Its chemical inertness prevents hydrolysis, corrosion, or enzymatic degradation, differentiating it from other polymers that may show deterioration in moist or inflammatory environments. This stability is particularly important in the maxillofacial region, where implants are exposed to the salivary microbiome, inflammatory cytokines, and continuous mechanical forces.

### Mechanical compatibility with craniofacial bone

One of the defining advantages of PEEK is its elastic modulus, which closely approximates cortical bone and thereby reduces the mismatch in stiffness observed with metal implants. The modulus of PEEK ranges from 3–4 GPa (gigapascal) – significantly lower than titanium's 110 GPa – yet within the functional range required for many craniofacial load-bearing applications.^7^ This similarity in elasticity allows for more physiologic transmission of stress across bone–implant interfaces, potentially reducing the incidence of bone resorption associated with stress shielding.

Carbon fibre-reinforced PEEK (CFR-PEEK) further improves mechanical behaviour by incorporating aligned carbon fibres, which increase tensile strength, fatigue resistance, and directional stiffness.^8^ These enhancements are particularly valuable in mandibular reconstruction, where implants must support substantial bending moments generated by mastication. Experimental studies have demonstrated that CFR-PEEK plates perform comparably to titanium plates in biomechanical fatigue tests, suggesting that composite PEEK systems could eventually replace metallic constructs in selected cases.^9^ A comparison of key mechanical properties among bone, PEEK, titanium, and zirconia is presented in Table 1.

**Table 1:** Comparative properties of bone, PEEK, titanium, and zirconia relevant to maxillofacial applications

**Property**	**Cortical bone**	**PEEK**	**Titanium (Ti6Al-4V)**	**Zirconia**
Elastic modulus (GPa)	~14	3-4	~110	~200
Density (g/cmÂ^3^)	~2.0	~1.3	~4.5	~6.0
Radiographic behaviour	Radiolucent	Radiolucent	Radiopaque	Radiopaque
Osseointegration	Biological	Surface-dependent	Predictable	Good (bone contact)
Brittleness	Low	Low	Low	High
Fatigue resistance	Physiological	High	High	Moderate
CAD/CAM compatibility	N/A	Excellent	Excellent	Excellent
CAD/CAM, computer-aided design/computer-aided manufacturing; GPa, gigapascal; N/A, not applicable; PEEK, polyetheretherketone; Ti6Al4V, titanium alloy				

### Biological interactions and cellular response

Despite its favourable mechanical profile, PEEK exhibits limited biological activity due to its hydrophobic and chemically inert surface. Cellular adhesion, protein adsorption, and osteointegration of PEEK are significantly lower compared with titanium, resulting in a tendency toward fibrous tissue encapsulation rather than direct bone bonding.^2^ While this characteristic contributes to its excellent biostability and low inflammatory potential, it poses major challenges for long-term stability in load-bearing applications.

Recent investigations have focused on modifying the PEEK surface to enhance cellular interactions. Surface roughening, plasma activation, chemical functionalisation, and bioactive nanocoatings have all demonstrated positive effects on osteoblast proliferation, mineralisation, and gene expression.^10^ These modifications aim to transform PEEK from a passive biomaterial into an osteoconductive platform capable of reliable fixation within craniofacial skeletal structures.

## Clinical applications of PEEK in oral and maxillofacial surgery

### Patient-specific implants for craniofacial reconstruction

The introduction of CAD/CAM technology and 3D printing has revolutionised craniofacial reconstruction by enabling highly precise patient-specific implant (PSI) fabrication. PEEK has become one of the materials best suited to this workflow because it can be milled or additively manufactured into complex shapes with minimal dimensional distortion.^11^ Such implants allow surgeons to restore facial symmetry and continuity with high accuracy, particularly in orbital, malar, frontal, and mandibular defects.

Clinical reports indicate that PEEK PSIs offer distinct advantages over titanium meshes, including superior fit, improved soft-tissue compatibility, and enhanced postoperative imaging.^12^ In orbital reconstruction, PEEK allows for restoration of orbital volume with minimal risk of sharp edges or unpredictable deformation.^13^ In mandibular cases, single-piece PEEK reconstructions eliminate the need for intraoperative bending, reducing operative time and improving contour accuracy.^14^ The material's lightweight structure also increases patient comfort and decreases the burden on compromised soft tissues.

### Reconstruction following oncologic resection

Head and neck oncologic resections often produce large three-dimensional defects requiring stable reconstruction that accommodates both functional and aesthetic requirements. PEEK's radiolucency provides a distinct advantage in these cases, as it allows for continuous oncologic surveillance using computerised tomography or magnetic resonance imaging without the interference created by metallic artefacts.^2^ This capability facilitates early detection of recurrence and enables accurate post-operative evaluation of adjacent structures.

In addition, PEEK's biomechanical properties support more even load distribution across reconstructed segments. When combined with vascularised bone grafts or particulate bone substitutes, PEEK frameworks can serve as scaffolds for biological integration.^15^ Early clinical evidence suggests that PEEK reconstructions exhibit favourable outcomes regarding speech, mastication, and facial symmetry, although long-term prospective data remain limited.^16^

### Osteosynthesis applications

The use of PEEK and CFR-PEEK plates in facial fracture management is an area of growing interest. These materials provide adequate mechanical stability while offering improved radiographic assessment capabilities. This advantage is especially useful in orbital or midface fractures, where post-operative imaging plays a central role in monitoring complication risks such as entrapment or implant malposition.

Biomechanical studies show that CFR-PEEK plates can resist cyclic loading similar to titanium plates, and their elasticity provides a more physiological stress environment for healing bone.^17^ Clinically, patients may experience reduced cold sensitivity and fewer palpability complaints due to the material's low thermal conductivity and softness relative to metals. However, screw fixation remains a technical challenge, as PEEK screw-holes are more prone to deformation or thread stripping, necessitating careful insertion torque control and possibly hybrid screw designs.^6^

### Dental implantology and prosthetic applications

Although PEEK has gained widespread acceptance in dental prosthetics – particularly for temporary abutments, provisional frameworks, and implant-supported superstructures – its role as a definitive dental implant material remains limited. This is primarily attributable to its intrinsically bioinert surface and the lack of predictable long-term osseointegration when compared with conventional implant materials. For this reason, dental and prosthetic applications of PEEK are addressed in this review in a restricted and contextual manner, focusing on current clinical use and biological limitations rather than broad comparisons with established dental implant biomaterials.

The lack of intrinsic osteointegration of PEEK limits long-term stability.^18^ Nonetheless, research into surface-treated PEEK implants has shown encouraging results. Techniques such as plasma treatment, HA nanoparticle coating, and nanotopographic modification improve bone-implant contact and may eventually support PEEK's clinical use in specific implantology scenarios.^19^^,^^20^

Prosthetically, PEEK's shock-absorbing capacity helps reduce occlusal stress transfer, making it ideal for immediate-loading protocols or patients with parafunctional habits. Its soft-tissue compatibility also promotes healthy peri-implant mucosa, reducing inflammation around healing abutments.^21^

### Temporomandibular joint prosthetics

The temporomandibular joint is one of the most mechanically complex joints in the human body, and its prosthetic replacement requires materials capable of withstanding high shear, compressive, and translational stresses. PEEK offers several advantages, including low friction, high fatigue resistance, and a physiologically compatible elastic modulus.^22^ These properties allow for more natural joint mechanics and reduced wear compared with traditional metallic components.

Emerging clinical evidence suggests that PEEK-based TMJ prostheses result in less postoperative discomfort and improved functional adaptation.^23^ PEEK's radiolucency further enables detailed postoperative imaging of surrounding bone structures, facilitating long-term follow-up. An overview of major clinical applications of PEEK in maxillofacial surgery is summarised in Table 2.

**Table 2:** Major clinical applications of PEEK in oral and maxillofacial surgery

**Application area**	**Clinical use**	**Key advantage**	**Key limitation**	**Clinical maturity**
Patient-specific implants	Craniofacial reconstruction	Precise fit, radiolucency	Cost	Emerging
Oncologic reconstruction	Segmental defects	Imaging follow-up	Limited integration	Emerging
Osteosynthesis	Fracture fixation	Bone-like elasticity	Screw sensitivity	Early clinical
Dental prosthetics	Abutments, frameworks	Shock absorption	Poor intrinsic integration	Established
TMJ prosthetics	Joint components	Low friction	Limited long-term data	Experimental
TMJ, temporomandibular joint				

## Limitations and challenges

The primary barrier to widespread adoption of PEEK in maxillofacial surgery is its limited ability to bond with bone. While inert surface of PEEK resists corrosion and degradation, it also restricts osteogenic cell attachment, resulting in fibrous encapsulation rather than osseous integration.^2^ This limitation is particularly problematic in load-bearing applications such as mandibular reconstruction or dental implants, where micromotion can provoke long-term instability.

Attempts to address this limitation through surface texturing alone have shown mixed results. While micro-roughening can improve mechanical interlocking of PEEK, meaningful osteointegration requires chemical activation or bioactive coatings that promote protein adsorption and osteoblast differentiation.^19^ Achieving consistent, durable, and clinically validated surface modifications remains a major research frontier.

PEEK exhibits lower resistance to thread stripping than titanium, which complicates screw fixation during osteosynthesis or reconstruction.^7^ Over-tightening or improper angulation can result in deformation of screw holes, compromising implant stability. CFR-PEEK improves screw retention but does not fully resolve this issue.^24^ Optimised screw designs, pre-threaded holes, or hybrid titanium–PEEK constructs may provide solutions, but standardised protocols have yet to be established.

Although PEEK offers significant imaging advantages, its radiolucency can occasionally be a limitation, particularly when surgeons require visualisation of the implant margins or screw positions. Techniques such as embedding radiopaque markers have been developed, yet their integration varies among implant systems. Soft-tissue interactions also require attention, as roughened surfaces may provoke irritation or promote bacterial adherence if not properly engineered.

Despite the growing enthusiasm for PEEK, robust long-term human data remain scarce. Most clinical studies on PEEK involve small cohorts or short follow-up durations, particularly in TMJ prosthetics and mandibular reconstruction.^21^ Randomised controlled trials comparing PEEK to titanium are essential for establishing evidence-based guidelines and ensuring safe integration into mainstream practice.

### Comparative perspective: PEEK versus titanium and zirconia

Within digitally planned and CAD/CAM-driven maxillofacial reconstruction, PEEK, titanium, and zirconia represent three fundamentally different material classes, each associated with distinct mechanical, biological, and clinical performance profiles. Titanium remains the most extensively validated material, offering excellent strength and long-term osseointegration; however, its high elastic modulus, radiopacity, and potential for imaging artefacts represent notable limitations in complex craniofacial and TMJ applications.^25^

Zirconia, as a ceramic and metal-free alternative, provides high strength, favourable aesthetics, and good biocompatibility, yet its intrinsic brittleness and limited tolerance to complex three-dimensional loading restrict its applicability in large patient-specific implants and load-bearing reconstructive scenarios.^26^

In contrast, PEEK offers a unique combination of bone-like elasticity, radiolucency, and compatibility with additive manufacturing and patient-specific design. While its mechanical behaviour is advantageous for stress distribution and imaging follow-up, its clinical performance is strongly dependent on surface modification strategies aimed at overcoming its bioinert nature.^27^ From a comparative perspective, PEEK should therefore be viewed not as a direct replacement for titanium or zirconia, but as a complementary material whose indications are defined by biomechanical context, digital workflow requirements, and biological optimisation strategies.

## Advances in surface modification

Plasma surface activation is among the most effective strategies for increasing PEEK's hydrophilicity. Oxygen plasma creates functional groups that enhance protein adsorption, while argon plasma roughens the surface at the nanoscale.^28^ These changes improve early-stage osteoblast adhesion and proliferation, although the long-term stability of plasma-induced modifications remains under investigation.

Nanotopography plays a critical role in modulating cell behaviour. Nanopores, nanogrooves, and nano-pits mimic the architecture of natural bone and can stimulate osteogenic differentiation. Studies demonstrate increased alkaline phosphatase activity, improved extracellular matrix mineralisation, and enhanced osteogenic gene expression on nanotextured PEEK surfaces.^29^ This emerging field shows considerable promise for achieving reliably osteoconductive PEEK implants.

Bioactive coatings such as hydroxyapatite, calcium phosphates, silica-based bio glasses, and collagen peptides significantly alter surface chemistry to promote mineral deposition and bone bonding. Techniques such as electrophoretic deposition, sol-gel processing, and particle embedding allow integration of osteoconductive particles into the PEEK matrix.^29^ Additionally, biomolecule immobilisation with growth factors such as BMP2 provides an osteoinductive dimension, accelerating bone formation around the implant.

Incorporating carbon fibres, ceramic fillers, or bioactive nanoparticles into PEEK's matrix creates composite materials with enhanced strength and improved biological activity. CFR-PEEK, in particular, demonstrates a mechanical performance profile suitable for high-load regions, while hybrid bioactive composites show superior osseointegration in animal studies.^30^ These composite strategies represent a major trajectory for next-generation PEEK biomaterials.

## Additive manufacturing and digital Integration

The advent of high-temperature additive manufacturing systems has transformed PEEK from a machinable material into one that can be additively manufactured into highly complex structures. Laser sintering and fused deposition modelling allow creation of porous scaffolds, integrated fixation features, and gradient-density implants that mimic bone's hierarchical structure.^31^ These capabilities not only enhance anatomical accuracy but also enable functional optimisation for load-bearing applications.

PEEK is particularly well suited to digital workflows. Advanced virtual surgical planning platforms allow surgeons and engineers to co-design implants tailored to the patient's anatomy, pathology, and biomechanical needs. Artificial intelligence (AI) further accelerates this process by automating segmentation, defect prediction, and implant optimisation. AI-driven design algorithms can analyse load distribution and suggest modifications that improve implant durability or reduce stress concentration.

PEEK's electrical insulating properties facilitate integration of embedded sensors capable of monitoring strain, temperature, infection biomarkers, and occlusal load patterns.^6^ These smart implants represent a paradigm shift in post-operative monitoring, allowing clinicians to detect complications at early stages, track healing progression, and personalise post-operative care. Early prototypes demonstrate feasibility in mandibular reconstruction and TMJ prosthetics, with future applications likely to expand into real-time feedback systems.

## Future perspectives

As surface engineering and composite technology advance, PEEK is expected to be increasingly adopted in metal-sensitive patients and those requiring refined aesthetic outcomes. Its comfort, lightweight nature, and imaging transparency align with modern reconstructive goals. Over time, PEEK may replace titanium in selected applications, particularly in PSIs and midface reconstruction.

Regenerative medicine is poised to transform large craniofacial defect management. Bioactive PEEK scaffolds seeded with mesenchymal stem cells, osteogenic biomolecules, or controlled-release growth factor systems offer the potential for hybrid constructs that combine mechanical stability with biological regeneration. These strategies may eventually produce scaffold-integrated reconstructions capable of restoring both skeletal structure and biological function.

PEEK's elasticity and low weight make it well suited for paediatric patients, in whom bone quality, growth potential, and limited tolerance for repeated surgeries pose unique challenges.^2^ Custom paediatric implants with modular or expandable configurations represent a future area of innovation.

To support broader clinical adoption, well-designed randomised trials are needed to compare PEEK and titanium across diverse conditions such as mandibular fractures, segmental reconstructions, orbital repairs, and TMJ replacement. Establishing clear indications and standardised surgical protocols will be critical for ensuring safe and effective use.

## Conclusion

PEEK represents a promising material for selected oral and maxillofacial and TMJ applications due to its bone-like elasticity, radiolucency, and compatibility with digital manufacturing workflows. However, its clinical performance remains constrained by limited intrinsic bioactivity and dependence on surface modification strategies to achieve reliable biological integration. While experimental studies report encouraging osteoconductive findings, robust long-term clinical evidence is still lacking. Future clinical adoption will therefore depend on well-designed studies confirming predictable biological integration alongside mechanical reliability.

## Data Availability

No new data were generated. All information is derived from previously published studies.
